# Assessment of hemoglobin-to-red cell distribution width ratio to predict all-cause mortality in patients with sepsis: a retrospective cohort study from the MIMIC-IV database

**DOI:** 10.3389/fmed.2026.1836373

**Published:** 2026-07-13

**Authors:** Zhiwei Su, Yin Wen, Wenhong Zhong, Hongguang Ding, Hongke Zeng

**Affiliations:** 1 Department of Critical Care Medicine, Guangdong Provincial People's Hospital, Guangdong Academy of Medical Sciences, Southern Medical University, Guangzhou, Guangdong, China; 2 Department of Emergency Medicine, Guangdong Provincial People's Hospital, Guangdong Academy of Medical Sciences, Southern Medical University, Guangzhou, Guangdong, China

**Keywords:** hemoglobin-to-red cell distribution width ratio, MIMIC-IV database, mortality, propensity score matching, sepsis

## Abstract

**Background:**

Over the past few years, the Hemoglobin-to-Red Cell Distribution Width Ratio (HRR) has garnered attention as a potential indicator of inflammatory and nutritional status. This study seeks to explore the link between HRR and the prognosis of sepsis patients.

**Methods:**

Relying on the Medical Information Mart for Intensive Care IV (MIMIC-IV) database, this retrospective cohort study delved into the clinical data of sepsis patients. Through X-tile, patients were categorized into Low-score and High-score groups. Propensity score matching (PSM) analysis was leveraged to control for confounding factors. The main focus was the 28-day mortality of sepsis patients. Kaplan-Meier curves were used to compare survival probabilities across groups. The Cox proportional hazards model and Restricted Cubic Spline were applied to scrutinize the relationship between HRR and 28-day mortality. The predictive capacity of HRR was assessed via ROC curve analysis and subgroup analysis.

**Results:**

The study encompassed 28,812 sepsis patients, with 5,697 (19.77%) succumbing to death within 28 days. Post-PSM analysis, the high-score group exhibited a mortality rate of 20.23%, markedly lower than the low-score group's 25.72% (*P* < 0.05). Within the unmatched cohort, Cox proportional hazards regression analysis also revealed that the High-score group had a reduced 28-day mortality risk (*HR*, 0.73; 95% *CI*, 0.68–0.79). The restricted regression curve demonstrated a non-linear relationship between HRR and the 28-day mortality rate of sepsis patients (*P* < 0.05). Threshold analysis identified 0.78 as the inflection point. When HRR was below 0.78, each one-unit rise in HRR corresponds to a 26% decrease in the 28-day mortality rate (*HR*, 0.26; 95% *CI*, 0.19–0.38). Combining HRR with disease severity scores enhances the predictive value for 28-day mortality.

**Conclusion:**

There's a significant negative correlation between HRR and the 28-day mortality rate of sepsis patients, making HRR an independent prognostic factor. Integrating HRR into existing scoring models can boost predictive accuracy.

## Introduction

Sepsis 3.0 defines sepsis as a life-threatening organ dysfunction caused by the host's dysfunctional response to infection (1). It's a prevalent critical condition in emergency departments and ICUs. In 2017, globally, 48.9 million sepsis cases were diagnosed, with 11 million fatalities, contributing 19.7% to global deaths and posing a significant health burden (2). Thus, finding effective, non-invasive, and readily available biomarkers to predict sepsis outcomes is vital. These markers can facilitate timely clinical decisions, improve patient recovery, and cut death rates.

Unlike established biomarkers such as the platelet-lymphocyte ratio (PLR), the neutrophil-to-lymphocyte ratio (NLR), and neutrophil-platelet ratio (NPR), the Hemoglobin-to-Red Cell Distribution Width Ratio (HRR) may be an independent risk factor for all-cause, cancer, and cardiovascular mortality (3–5). HRR combines factors with hemoglobin and red Cell Distribution Width (RDW) offering a comprehensive measure of a patient's systemic inflammatory response and nutritional health. Furthermore, there is evidence suggesting that HRR can be used to assess the prognosis of conditions such as chronic kidney disease, rheumatoid arthritis, and neurological disorders (6–8).

Given the pivotal role of nutrition and inflammation in sepsis progression, lower HRR might be associated with a higher risk of death. This study focuses on the association between HRR and sepsis prognosis in the ICU setting, aiming to offer new viewpoints on factors influencing clinical outcomes and to identify potential strategies to boost patient welfare and survival chances.

## Methods

### Data sources

The data utilized in this study originates from the MIMIC-IV 3.0 database (9), a collaborative effort between Beth Israel Deaconess Medical Center (BIDMC) and Massachusetts Institute of Technology (MIT). This freely accessible database encompasses over 50,000 ICU admissions from BIDMC between 2008 and 2019. This study was conducted using the publicly available MIMIC-IV 3.0 database. The Institutional Review Boards of MIT and BIDMC approved the database creation and granted a waiver for informed consent. As the data are de-identified and publicly available, additional patient consent was not required for this retrospective analysis. The lead author, ZhiWei Su, gained database access by completing the CITI program and associated exams (Record ID: 68674529). Clinical trial number: not applicable.

### Study population

This study included adult critically ill patients with sepsis, defined as suspected or documented infection with an acute increase of at least 2 in the total Sequential Organ Failure Assessment (SOFA) score (1). Excluded were patients under 18 years of age, those with an ICU stay of less than 24 h, or those without recorded hemoglobin and RDW values on the first day of admission. For patients with multiple ICU admissions, only the records of the first admission were analyzed.

### Variable selection

**Figure 1:**
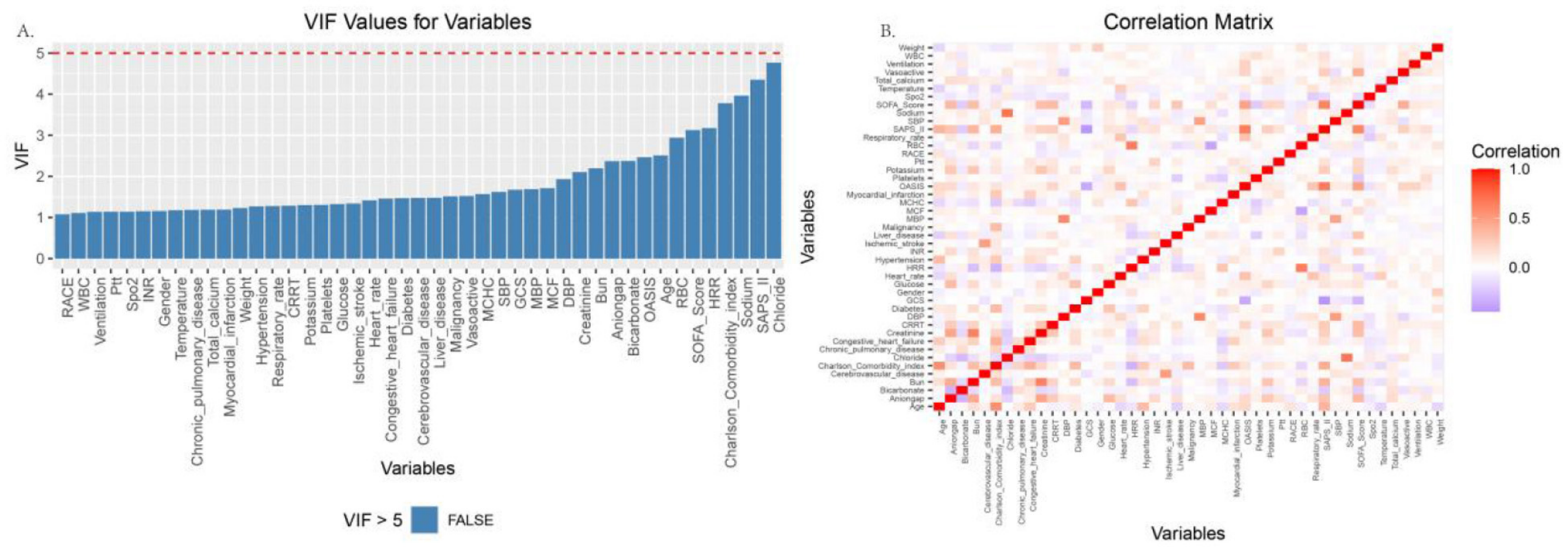
The VIF values of the variables and the analysis of the correlation matrix. **(A)** The VIF values of each variable. **(B)** Correlation matrix between variables.

Data were extracted using PostgreSQL software (version 12.19.2) and Navicat Premium software (version 15) through structured query language (SQL). These data included baseline demographic variables, vital signs, comorbidities, disease scores, therapeutic interventions, and laboratory tests. The baseline demographic variables included gender, weight, age and race. The vital signs included the mean blood pressure (MBP), heart rate, Systolic Blood Pressure (SBP), Diastolic Blood Pressure (DBP), Temperature, SpO_2_, and respiratory rate (RR). The comorbidities included Myocardial infarction, Liver disease, Malignancy, Ischemic stroke, Hypertension, diabetes, Congestive heart failure, Chronic pulmonary disease, and Cerebrovascular disease. Disease scores included the Charlson Comorbidity Index (CCI), A New Simplified Acute Physiology Score (SAPS II), Glasgow Coma Scale (GCS), Oxford acute severity of illness score (OASIS) and SOFA Score, HRR exhibited a non-linear relationship with 28-day mortality. An elevated HRR is associated with reduced 28-day, 60-day, 90-day, and in-hospital mortality among sepsis patients. Therapeutic interventions included mechanical ventilation, vasopressor use, and circulation renal replacement treatment (CRRT). Vasopressor use was identified as any application of vasopressin, dopamine, dobutamine, norepinephrine, or epinephrine throughout the ICU stay. The laboratory tests included hemoglobin, Platelets, red cell volume distribution width (RDW), white blood cell count (WBC), chloride, red blood cell count (RBC), Creatinine, blood urea nitrogen (BUN), potassium, glucose, sodium, Bicarbonate, Aniongap, Total calcium, partial thromboplastin time (PTT), international normalized ratio (INR), Mean corpuscular hemoglobin concentration (MCHC), Mean Corpuscular Volume (MCV). The indicators above were collected during the initial 24 h of ICU admission. For major variables (hemoglobin and RDW), the first recorded value was used. For the remaining indicators, the worst value that was most relevant to the severity of the disease was taken when there were multiple recordings during the initial 24 h following ICU admission. The formula provided was used to determine the HRR = hemoglobin (g/dl)/RDW (%) (10). Variables exhibiting over 20% missing values were removed, while the missing values of the rest of the variables were imputed with Multiple Imputation (11). To assess multicollinearity among variables, the variance inflation factor (VIF) was utilized, with a value of *VIF* ≥ 5 signifying the existence of multicollinearity (12). Table 1 shows the variables that were removed due to the presence of more than 20% missing values and the issue of multicollinearity. Figure 1 shows that the VIF values of the variables and the analysis of the correlation matrix.

**Table 1:** The missing variables are due to a missing value rate greater than 20% or multicollinearity.

The data is missing by more than 20%	BMI, Height, Albumin, Globulin, Total_Protein, Basophils, Eosinophils, Lymphocytes, Monocytes, Neutrophils, Atypically_mphocytes, Immature_Granulocytes, Metamyelocytes, Nrbc, Ddimer, Fibrinogen, Alt, Alp, Ast, Bilirubin_Total, Bilirubin_Direct, Bilirubin_Indirect, CKCPk, CKMB, LDH, PO_2_, PCO_2_, PaO_2_, FiO_2_ ratio, PH, Base excess, Bicarbonate, Chloride, Free_Calcium, Potassium, Sodium, Lactate, TG, TC, LDL, HDL, CRP
VIF is greater than 5 or the tolerance is less than 0.1	PT, APSIII, MCH

### Outcomes

The primary outcome was 28-day all-cause mortality. Secondary outcomes included mortality rates at 60 days and 90 days, in-hospital mortality, and in-ICU mortality (both measured in days).

### Statistical analysis

In the analytical approach of this study, continuous variables were delineated by their median values accompanied by the interquartile range (IQR), with their disparities evaluated through either the *t*-test or the Mann–Whitney *U*-test. Before applying the Mann–Whitney *U*-test, we tested the distribution of the continuous variables using the Shapiro–Wilk test for normality. Given that the variables did not follow a normal distribution, the Mann–Whitney *U*-test was deemed appropriate. For categorical variables, presentations involved enumerations alongside proportions, and their comparisons were facilitated by either the Chi-square test or Fisher's exact test.

We set a stringent criterion and excluded variables with over 20% missing values. The remaining variables with missing data was imputed by Multiple Imputation. To handle the missing data in the variables (the specific missing variables can be seen in Supplementary Figure 1), we employed a multiple imputation technique based on chained equations. All operations were completed using the mice package in R software. We assumed that the data met the condition of random missing (MAR). A total of *m* = 5 complete imputed data sets were generated. The imputation model included all the analysis variables, the outcome variables (28-day mortality rate and survival time within 28 days), and the HRR variables. For continuous variables, the predictive mean matching (pmm) method was used for imputation, and for binary variables, the logistic regression (logreg) method was used. Each imputation model was diagnosed to ensure convergence and the rationality of the imputed values. After independently fitting the COX proportional regression model on each imputed data set, the final results were combined according to the Rubin rule (as Supplementary Tables 1, 2 and Supplementary Figures 2, 3 for details).

The baseline characteristics between 28-day survivors and non-survivors were compared. The optimal HRR cutoff was determined using X-tile software by systematically evaluating all potential values via log-rank tests to maximize survival separation. The threshold of 0.6 divided patients into Low-score (< 0.6) and High-score (≥0.6) groups (13). We acknowledge that data-driven cutoff selection within the same dataset may introduce overfitting and optimistic bias. Therefore, we additionally performed analyses using: (1) HRR as a continuous variable, (2) clinically meaningful cutoffs, and (3) quintiles to assess the robustness of findings. External validation in an independent cohort is warranted before clinical application of the 0.6 cutoff. Kaplan–Meier survival curves and the log-rank test were used to assess survival differences among the two groups. Propensity score matching (PSM) analysis was performed to minimize baseline differences between the two groups, using one-to-one matching with a caliper width of 0.05 to ensure precise matching (14). The effectiveness of PSM was assessed using the standardized mean difference (SMD), with SMD values < 0.1 indicating no significant differences (15). Hemoglobin and RDW were intentionally excluded from the propensity model as they directly constitute the HRR exposure variable. We acknowledge that this approach may leave residual imbalance in underlying hematologic status between groups. Variables with large standardized mean differences (*SMD* > 0.1) before matching, including RBC count, BUN, liver disease, malignancy, and congestive heart failure, were monitored closely. Balance was assessed using SMD, with values < 0.1 indicating adequate balance after matching.

To assess multicollinearity among variables, the variance inflation factor (VIF) was utilized, with a value of VIF ≥ 5 signifying the existence of multicollinearity (12). The independent association between the HRR and 28-day mortality in septic patients were evaluated using Cox proportional hazard models. Five models were constructed: Model 1: Crude. Model 2: Adjust: Gender, RACE, Age, Weight, Heart rate, SBP, DBP, MBP, RR, Temperature, SpO_2_. Model 3: further adjusted for WBC, Platelets, RBC, Creatinine, Bun, Glucose, Aniongap, Bicarbonate, Sodium, Total calcium, Chloride, Potassium, INR, PTT, MCHC, MCV. Model 4: further adjusted for Vsoactive, Ventilation, CRRT, SAPSII, GCS, OASIS, SOFA Score, Charlson Comorbidity index. Model 5: further adjusted for Myocardial infarction, Liver disease, Malignancy, Ischemic stroke, Hypertension, Diabetes, Congestive heart failure, Chronic pulmonary disease, Cerebrovascular disease. And the corresponding *E*-values were measured as well (16). A two-segment linear regression model, which was based on restrictive regression curve, were applied to evaluate the non-linear association and threshold effect between the HRR and 28-day mortality. To evaluate the prognostic value of the HRR, we performed ROC curve analysis to calculate the area under the curve (AUC) values and compared them using the DeLong-test. We also assessed the incremental predictive value of the HRR by combining it with severity of illness scores, including SAPSII, OASIS, SOFA Score, and Charlson Comorbidity index. Additionally, we performed a subgroup analysis in sepsis patients to compare whether there is consistency or not.

Due to its high correlation with SAPSII, APS-III was excluded from Model 4. Variables with more than 20% missing values (lactate, C-reactive protein, procalcitonin, albumin, etc.) were also excluded from the main model.

The data analysis was conducted using R statistical software (version 4.2.2), SPSS Statistics 26, and GraphPad Prism 8, ensuring a comprehensive statistical evaluation. A two-tailed test approach was adopted, and *P* < 0.05 was considered statistically significant.

## Results

### The process of screening the research population

**Figure 2:**
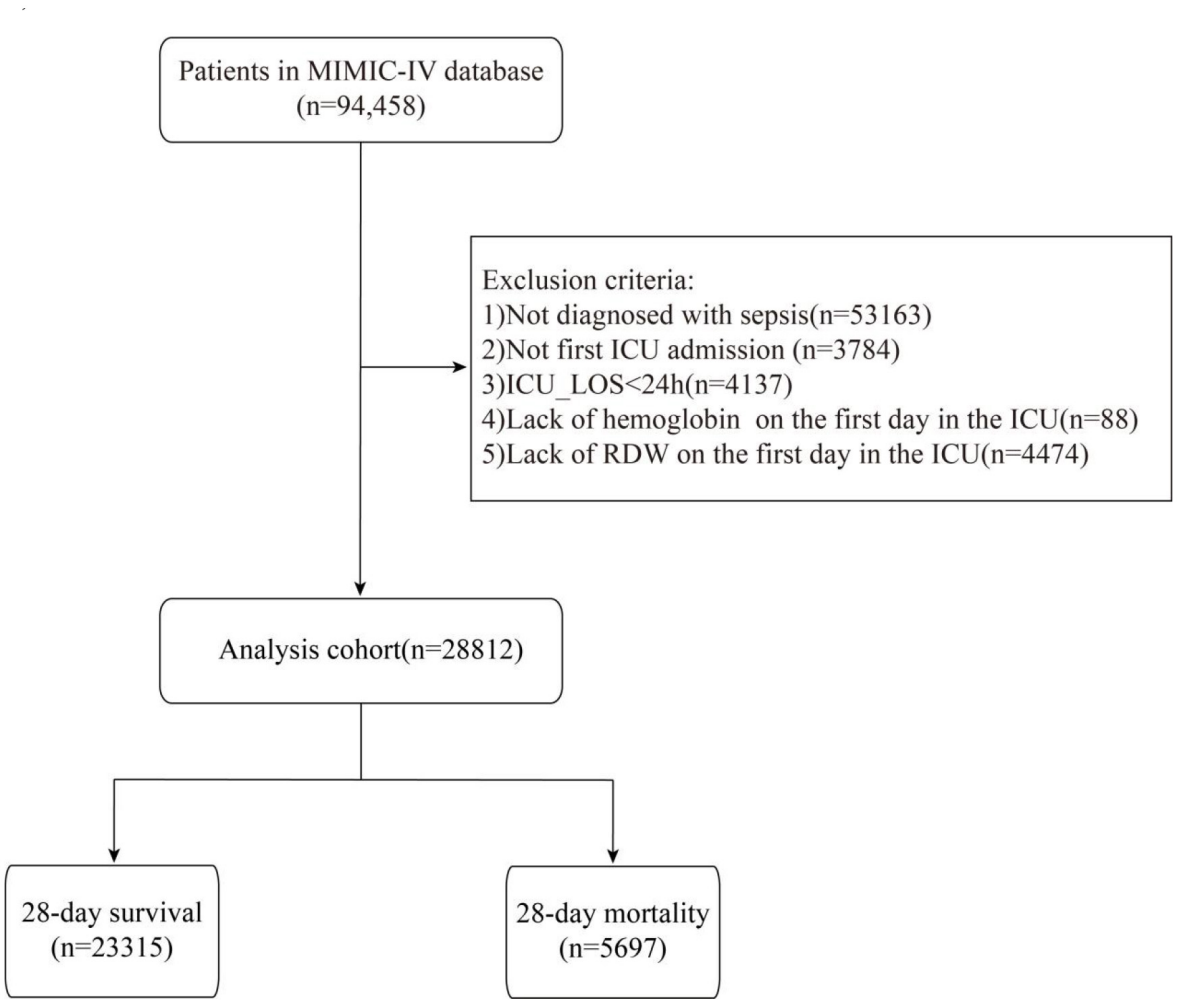
Flowchart of select study population from the MIMIC-IV database.

The study population was selected based on the sepsis-3.0 criteria. Initially, the MIMIC-IV database encompassed 94,458 patients. Subsequently, 53,163 non-septic patients and 3,784 non-first-time ICU admissions were excluded. Also, we eliminated 4,137 patients with ICU stays under 24 h, 88 patients missing hemoglobin records on the first ICU day, and 4,474 patients without RDW records on the first ICU day. In the end, the analysis covered 28,812 septic patients, including 23,115 28-day survivors and 5,697 28-day non-survivors (Figure 2).

### Baseline characteristics

We compared the baseline characteristics of the 28-day survivor and non-survivor groups (Table 2). The 28-day survivor group had higher levels of HRR, weight, SBP, temperature, SpO_2_, hemoglobin, platelets, RBC, bicarbonate, chloride, and MCHC. In contrast, the 28-day non-survivor group had lower age and higher heart rate, DBP, respiratory rate, WBC, creatinine, BUN, RDW, glucose, aniongap, total calcium, potassium, INR, PTT, and MCV. The non-survivor group also had higher severity of illness scores (SAPSII, SOFA, CCI, OASIS), higher proportions of comorbidities (myocardial infarction, liver disease, malignancy, ischemic stroke, hypertension, diabetes, congestive heart failure, chronic pulmonary disease, cerebrovascular disease), and higher rates of interventions like CRRT, vasopressors, and ventilation.

**Table 2:** Baseline characteristics according to survivors and non-survivors groups.

Variables	Total (*n* = 28,812)	Survivor (*n* = 23,115)	Non-survivor (*n* = 5,697)	*P*
Gender [male, *n* (%)]	16,838 (58.44)	13,617 (58.91)	3,221 (56.54)	0.001
RACE [white, *n* (%)]	19,114 (66.34)	15,588 (67.44)	3,526 (61.89)	< 0.001
Age (years)	67.85 (56.93, 78.30)	66.81 (56.00, 77.05)	72.90 (61.48, 82.67)	< 0.001
Weight (kg)	79.70 (66.80, 95.20)	80.25 (67.80, 96.10)	76.00 (63.40, 91.00)	< 0.001
Heart rate (bpm)	104.00 (91.00, 119.00)	103.00 (90.00, 118.00)	110.00 (95.00, 126.00)	< 0.001
SBP (mmHg)	144.00 (131.00, 160.00)	145.00 (132.00, 160.00)	143.00 (128.00, 159.00)	< 0.001
DBP (mmHg)	84.00 (73.00, 97.00)	84.00 (73.00, 97.00)	86.00 (74.00, 99.00)	< 0.001
MBP (mmHg)	100.00 (90.00, 114.00)	100.00 (90.00, 113.00)	100.00 (89.00, 115.00)	0.753
Respiratory rate (/min)	28.00 (24.00, 32.00)	27.00 (24.00, 32.00)	30.00 (25.00, 34.00)	< 0.001
Temperature (°C)	37.33 (37.00, 37.90)	37.33 (37.00, 37.90)	37.22 (36.89, 37.83)	< 0.001
SpO_2_ (%)	92.00 (90.00, 95.00)	93.00 (90.00, 95.00)	91.00 (88.00, 94.00)	< 0.001
Hemoglobin (g/dl)	11.00 (9.60, 12.60)	11.10 (9.70, 12.70)	10.60 (9.10, 12.40)	< 0.001
WBC ( × 10^9^)	13.90 (9.90, 19.00)	13.70 (9.80, 18.60)	14.90 (10.30, 20.90)	< 0.001
Platelets ( × 10^9^)	201.00 (144.00, 276.00)	201.00 (146.00, 273.00)	200.00 (129.00, 288.00)	< 0.001
RBC ( × 109)	3.41 (2.99, 3.89)	3.44 (3.02, 3.89)	3.31 (2.86, 3.85)	< 0.001
Creatinine (mg/dl)	1.20 (0.90, 2.00)	1.10 (0.80, 1.80)	1.60 (1.00, 2.70)	< 0.001
BUN (mg/dl)	24.00 (16.00, 41.00)	22.00 (15.00, 37.00)	35.00 (22.00, 56.00)	< 0.001
RDW	15.00 (13.80, 16.80)	14.80 (13.70, 16.40)	16.10 (14.60, 18.20)	< 0.001
Glucose (mg/dl)	147.00 (119.00, 199.00)	144.00 (117.00, 192.00)	163.00 (127.00, 223.00)	< 0.001
Aniongap (mEq/L)	16.00 (13.00, 19.00)	15.00 (13.00, 18.00)	18.00 (15.00, 22.00)	< 0.001
Bicarbonate (mEq/L)	24.00 (21.00, 27.00)	24.00 (22.00, 27.00)	23.00 (20.00, 26.00)	< 0.001
Sodium (mEq/L)	140.00 (137.00, 143.00)	140.00 (137.00, 142.00)	140.00 (136.00, 144.00)	0.062
Total calcium (mg/dl)	8.50 (8.00, 9.00)	8.50 (8.00, 9.00)	8.60 (8.10, 9.10)	< 0.001
Chloride (mEq/L)	106.00 (102.00, 110.00)	107.00 (103.00, 110.00)	105.00 (100.00, 110.00)	< 0.001
Potassium (mEq/L)	4.50 (4.10, 5.00)	4.50 (4.10, 5.00)	4.60 (4.20, 5.30)	< 0.001
INR	1.40 (1.20, 1.70)	1.40 (1.20, 1.60)	1.50 (1.30, 2.20)	< 0.001
PTT (s)	33.70 (29.00, 45.30)	33.10 (28.80, 42.80)	37.70 (30.20, 57.80)	< 0.001
MCHC (g/dl)	33.10 (31.90, 34.20)	33.20 (32.00, 34.30)	32.70 (31.50, 33.90)	< 0.001
MCV (fl)	91.00 (87.00, 96.00)	91.00 (87.00, 95.00)	93.00 (88.00, 98.00)	< 0.001
HRR	0.73 (0.59, 0.88)	0.76 (0.61, 0.89)	0.65 (0.52, 0.82)	< 0.001
SAPSII	39.00 (31.00, 49.00)	37.00 (30.00, 46.00)	49.00 (39.00, 59.00)	< 0.001
GCS	15.00 (13.00, 15.00)	15.00 (14.00, 15.00)	15.00 (12.00, 15.00)	< 0.001
OASIS	34.00 (28.00, 40.00)	33.00 (28.00, 38.00)	38.00 (32.00, 44.00)	< 0.001
SOFA score	6.00 (4.00, 8.00)	5.00 (3.00, 8.00)	8.00 (5.00, 11.00)	< 0.001
Charlson comorbidity index	5.00 (3.00, 7.00)	5.00 (3.00, 7.00)	7.00 (5.00, 9.00)	< 0.001
Vasoactive, *n* (%)	12,860 (44.63)	10,096 (43.68)	2,764 (48.52)	< 0.001
Ventilation, *n* (%)	26,183 (90.88)	20,781 (89.90)	5,402 (94.82)	< 0.001
CRRT, *n* (%)	2,024 (7.02)	1,042 (4.51)	982 (17.24)	< 0.001
Myocardial infarction, *n* (%)	5,368 (18.63)	4,109 (17.78)	1,259 (22.10)	< 0.001
Liver disease, *n* (%)	4,833 (16.77)	3,452 (14.93)	1,381 (24.24)	< 0.001
Malignancy, *n* (%)	3,909 (13.57)	2,654 (11.48)	1,255 (22.03)	< 0.001
Ischemic stroke, *n* (%)	1,131 (3.93)	752 (3.25)	379 (6.65)	< 0.001
Hypertension, *n* (%)	19,003 (65.96)	15,231 (65.89)	3,772 (66.21)	0.650
Diabetes, *n* (%)	9,449 (32.80)	7,553 (32.68)	1,896 (33.28)	0.384
Congestive heart failure, *n* (%)	9,531 (33.08)	7,227 (31.27)	2,304 (40.44)	< 0.001
Chronic pulmonary disease, *n* (%)	7,818 (27.13)	6,132 (26.53)	1,686 (29.59)	< 0.001
Cerebrovascular disease, *n* (%)	4,140 (14.37)	3,036 (13.13)	1,104 (19.38)	< 0.001
LOS hospital	8.26 (5.12, 15.20)	8.74 (5.26, 16.34)	6.91 (3.64, 12.14)	< 0.001
LOS ICU	3.27 (1.90, 6.81)	3.09 (1.83, 6.25)	4.38 (2.30, 8.35)	< 0.001

**Figure 3:**
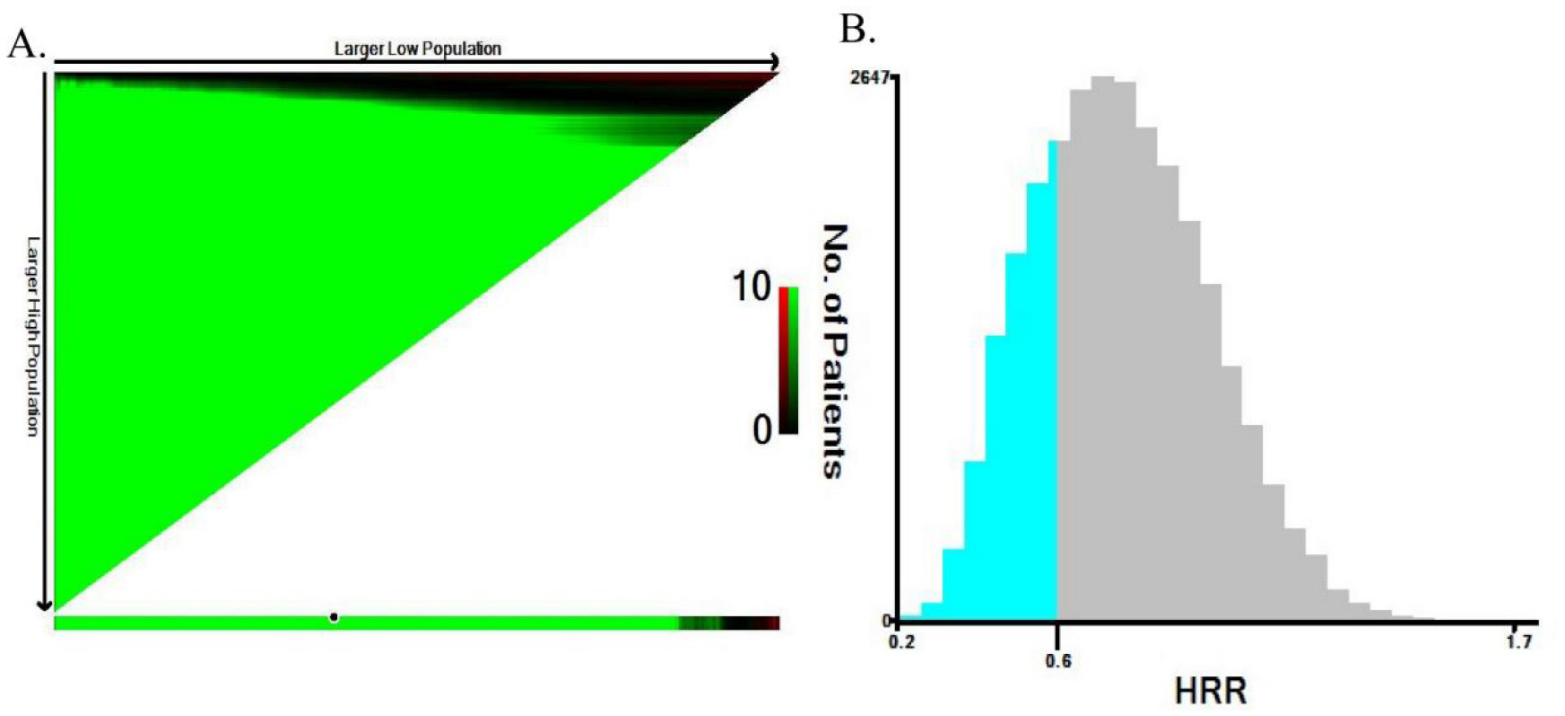
The optimal cutoff value. **(A)** Histograms. **(B)** X-tile plots.

**Figure 4:**
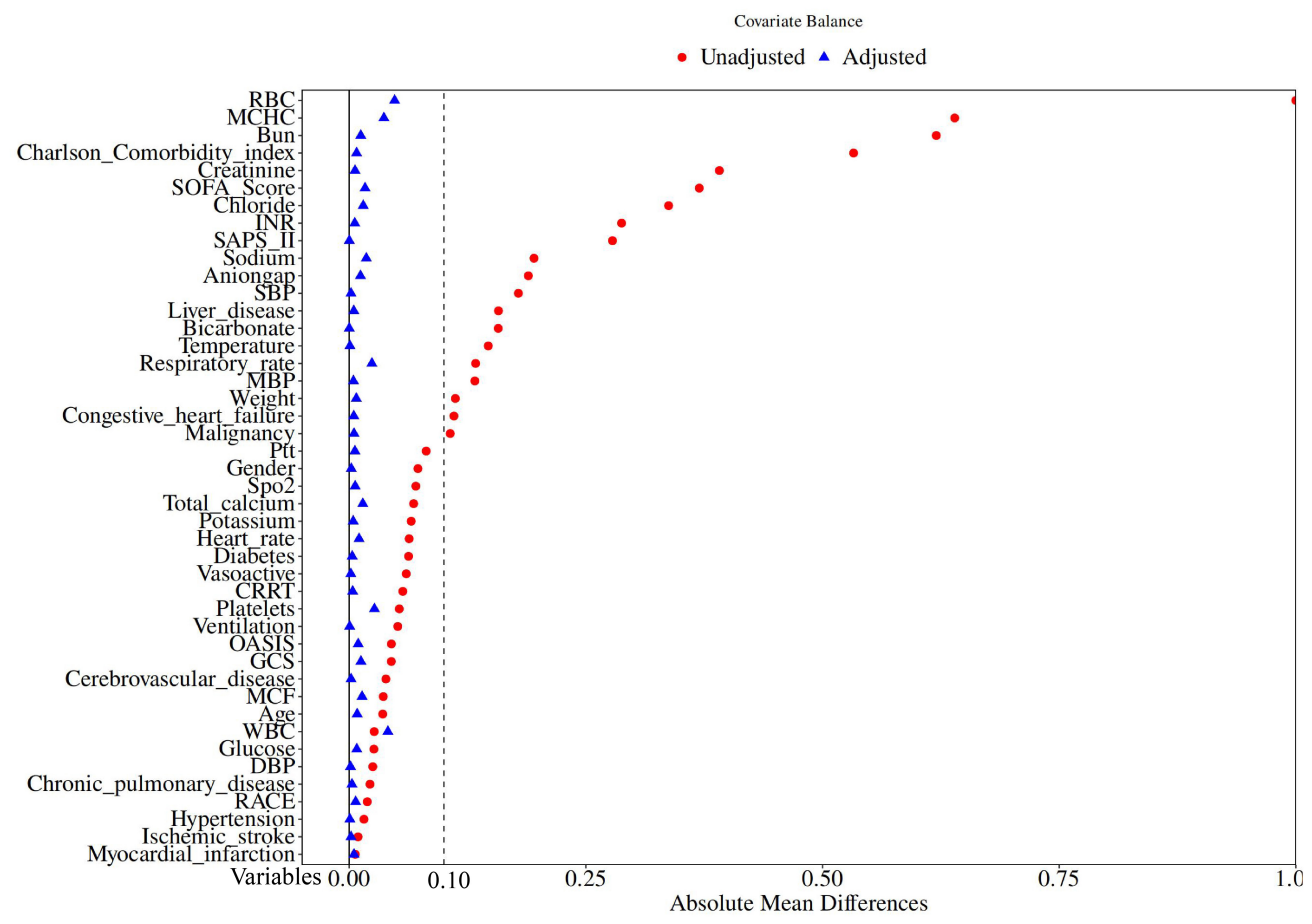
Tendency-based matching of changes in SMD before and after.

The optimal cutoff value of HRR (0.6) was identified via X-tile software (Figure 3), separating patients into Low-score (< 0.6) and High-score (≥0.6) groups. After one-to-one propensity score matching, each group consisted of 4,152 patients. Table 3 illustrates the baseline characteristics of the Low-score and High-score groups in both the pre-matched and post-matched cohorts. No statistically significant differences in baseline characteristics were observed after matching compared to the pre-matched cohort (all SMDs < 0.1). However, elements such as hemoglobin and RDW that directly contribute to the HRR were not considered as matching variables. The effectiveness of the matching process was assessed by calculating the standardized mean difference (SMD), with SMD values < 0.1 indicating no significant differences, both before and after PSM, as shown in Figure 4. The distribution of the Low-score and High-score groups became more balanced following the matching process (Supplementary Figure 4).

**Table 3:** Baseline demographic and clinical characteristics of low-score and high-score groups in the pre-matched and post-matched cohorts.

Variables	Before PSM				After PSM			
	Total (*n* = 28,812)	Low-score (<0.6) *n* = 7,875	High-score (≥0.6) *n* = 20,937	SMD	Total (*n* = 8,304)	Low-score (<0.6) *n* = 4,152	High-score (≥0.6) *n* = 4,152	SMD
Gender [male, *n* (%)]	16,838 (58.44)	4,187 (53.17)	12,651 (60.42)	0.148	4,349 (52.37)	2,179 (52.48)	2,170 (52.26)	−0.004
RACE [white, *n* (%)]	19,114 (66.34)	5,115 (64.95)	13,999 (66.86)	0.041	5,546 (66.79)	2,759 (66.45)	2,787 (67.12)	0.014
Age (years)	67.85 (56.93, 78.30)	67.92 (57.13, 78.31)	67.83 (56.87, 78.28)	−0.035	68.81 (58.24, 79.15)	68.54 (58.23, 79.08)	69.21 (58.27, 79.28)	−0.009
Weight (kg)	79.70 (66.80, 95.20)	77.20 (64.50, 92.80)	80.30 (67.75, 96.00)	0.112	76.62 (64.50, 92.00)	76.54 (64.50, 91.30)	76.70 (64.40, 92.50)	0.008
Vital signs								
Heart rate (bpm)	104.00 (91.00, 119.00)	106.00 (92.00, 121.00)	103.00 (91.00, 119.00)	−0.063	105.00 (91.00, 120.00)	105.00 (91.00, 120.00)	105.00 (91.00, 120.00)	0.01
SBP (mmHg)	144.00 (131.00, 160.00)	141.00 (128.00, 157.00)	145.00 (132.00, 161.00)	0.179	144.00 (130.00, 159.00)	144.00 (130.00, 159.00)	144.00 (130.00, 159.00)	0.002
DBP (mmHg)	84.00 (73.00, 97.00)	84.00 (73.00, 97.00)	84.00 (73.50, 97.00)	0.025	83.00 (73.00, 97.00)	84.00 (73.00, 97.00)	83.00 (72.00, 97.00)	−0.001
MBP (mmHg)	100.00 (90.00, 114.00)	98.00 (88.00, 112.00)	101.00 (91.00, 115.00)	0.133	99.00 (89.00, 113.00)	99.00 (89.00, 112.12)	99.00 (89.00, 113.00)	0.004
Respiratory rate (/min)	28.00 (24.00, 32.00)	28.00 (24.00, 33.00)	27.00 (24.00, 32.00)	−0.133	28.00 (24.00, 32.00)	28.00 (24.00, 32.00)	28.00 (24.00, 32.00)	0.023
Temperature (°C)	37.33 (37.00, 37.90)	37.22 (36.94, 37.72)	37.39 (37.00, 37.94)	0.147	37.28 (36.94, 37.80)	37.28 (36.94, 37.80)	37.28 (37.00, 37.80)	0.001
SpO_2_ (%)	92.00 (90.00, 95.00)	92.00 (89.00, 95.00)	93.00 (90.00, 95.00)	0.07	92.00 (90.00, 95.00)	92.00 (90.00, 95.00)	92.00 (90.00, 95.00)	−0.006
Laboratory tests								
WBC ( × 10^9^)	13.90 (9.90, 19.00)	12.90 (8.50, 18.90)	14.20 (10.50, 19.00)	−0.026	13.50 (9.20, 19.20)	12.90 (8.60, 18.80)	14.00 (10.00, 19.50)	0.036
Platelets ( × 10^9^)	201.00 (144.00, 276.00)	190.00 (112.00, 289.00)	204.00 (151.00, 272.00)	0.053	201.00 (135.00, 292.00)	199.00 (126.75, 293.00)	203.00 (142.00, 290.00)	0.023
RBC ( × 10^9^)	3.41 (2.99, 3.89)	2.88 (2.60, 3.23)	3.60 (3.23, 4.04)	1.147	3.09 (2.81, 3.40)	3.06 (2.76, 3.40)	3.11 (2.86, 3.40)	0.061
Creatinine (mg/dl)	1.20 (0.90, 2.00)	1.60 (1.00, 2.90)	1.10 (0.80, 1.70)	−0.391	1.40 (0.90, 2.50)	1.40 (0.90, 2.50)	1.40 (0.90, 2.50)	0.005
Bun (mg/dl)	24.00 (16.00, 41.00)	35.00 (21.00, 57.00)	22.00 (15.00, 35.00)	−0.62	30.00 (18.00, 49.00)	31.00 (19.00, 49.00)	29.00 (18.00, 49.00)	−0.01
Glucose (mg/dl)	147.00 (119.00, 199.00)	147.00 (117.00, 198.00)	147.00 (119.00, 199.00)	0.026	148.00 (119.00, 202.00)	147.00 (118.00, 198.00)	149.00 (120.00, 206.00)	0.009
Aniongap (mEq/l)	16.00 (13.00, 19.00)	17.00 (14.00, 20.00)	16.00 (13.00, 19.00)	−0.189	16.00 (13.00, 20.00)	16.00 (14.00, 20.00)	16.00 (13.00, 20.00)	0.011
Bicarbonate (mEq/L)	24.00 (21.00, 27.00)	23.00 (20.00, 27.00)	24.00 (22.00, 27.00)	0.157	24.00 (21.00, 27.00)	24.00 (21.00, 27.00)	24.00 (21.00, 26.00)	0
Sodium (mEq/L)	140.00 (137.00, 143.00)	139.00 (136.00, 142.00)	140.00 (138.00, 143.00)	0.195	139.00 (137.00, 142.00)	139.00 (136.00, 143.00)	139.00 (137.00, 142.00)	−0.017
Total calcium (mg/dl)	8.50 (8.00, 9.00)	8.40 (8.00, 9.00)	8.50 (8.10, 9.00)	0.068	8.50 (8.00, 9.00)	8.40 (8.00, 8.90)	8.50 (8.00, 9.00)	0.015
Chloride (mEq/L)	106.00 (102.00, 110.00)	104.00 (100.00, 109.00)	107.00 (103.00, 111.00)	0.337	106.00 (101.00, 110.00)	106.00 (101.00, 110.00)	106.00 (101.00, 110.00)	−0.014
Potassium (mEq/L)	4.50 (4.10, 5.00)	4.60 (4.10, 5.20)	4.50 (4.10, 5.00)	−0.065	4.60 (4.10, 5.10)	4.60 (4.10, 5.10)	4.60 (4.20, 5.10)	−0.004
INR	1.40 (1.20, 1.70)	1.50 (1.30, 2.10)	1.30 (1.20, 1.60)	−0.288	1.40 (1.20, 1.90)	1.50 (1.20, 2.00)	1.40 (1.20, 1.80)	−0.004
PTT (s)	33.70 (29.00, 45.30)	35.50 (29.90, 48.80)	33.10 (28.70, 43.90)	−0.081	34.50 (29.30, 47.00)	34.80 (29.60, 47.30)	34.10 (29.10, 46.50)	0.006
MCHC (g/dl)	33.10 (31.90, 34.20)	32.30 (31.00, 33.60)	33.30 (32.30, 34.40)	0.64	32.70 (31.50, 33.90)	32.70 (31.40, 34.00)	32.70 (31.70, 33.70)	0.037
MCV (fl)	91.00 (87.00, 96.00)	92.00 (86.00, 97.00)	91.00 (87.00, 95.00)	−0.036	91.00 (87.00, 96.00)	92.00 (86.00, 97.00)	91.00 (87.00, 96.00)	−0.013
Severity of illness scores								
SAPSII	39.00 (31.00, 49.00)	42.00 (34.00, 52.00)	38.00 (31.00, 48.00)	−0.278	41.00 (33.00, 50.00)	41.00 (33.00, 50.00)	41.00 (33.00, 50.00)	0
GCS	15.00 (13.00, 15.00)	15.00 (13.00, 15.00)	15.00 (13.00, 15.00)	−0.044	15.00 (13.00, 15.00)	15.00 (13.00, 15.00)	15.00 (14.00, 15.00)	0.014
OASIS	34.00 (28.00, 40.00)	34.00 (28.00, 40.00)	34.00 (28.00, 40.00)	−0.045	34.00 (28.00, 40.00)	34.00 (28.00, 40.00)	34.00 (29.00, 40.00)	−0.009
SOFA score	6.00 (4.00, 8.00)	6.00 (4.00, 9.00)	5.00 (3.00, 8.00)	−0.37	6.00 (4.00, 9.00)	6.00 (4.00, 9.00)	6.00 (4.00, 9.00)	−0.016
Charlson comorbidity index	5.00 (3.00, 7.00)	6.00 (4.00, 8.00)	5.00 (3.00, 7.00)	−0.533	6.00 (4.00, 8.00)	6.00 (4.00, 8.00)	6.00 (4.00, 8.00)	−0.007
Therapy, *n* (%)								
Vasoactive	12,860 (44.63)	3,170 (40.25)	9,690 (46.28)	0.121	3,593 (43.27)	1,793 (43.18)	1,800 (43.35)	0.003
Ventilation	26,183 (90.88)	6,863 (87.15)	19,320 (92.28)	0.192	7,414 (89.28)	3,706 (89.26)	3,708 (89.31)	0.002
CRRT	2,024 (7.02)	877 (11.14)	1,147 (5.48)	−0.249	685 (8.25)	335 (8.07)	350 (8.43)	0.013
Comorbidity diseases, *n* (%)								
Myocardial infarction	5,368 (18.63)	1,431 (18.17)	3,937 (18.80)	0.016	1,591 (19.16)	785 (18.91)	806 (19.41)	0.013
Liver disease	4,833 (16.77)	2,223 (28.23)	2,610 (12.47)	−0.477	1,798 (21.65)	909 (21.89)	889 (21.41)	−0.012
Malignancy	3,909 (13.57)	1,679 (21.32)	2,230 (10.65)	−0.346	1,463 (17.62)	721 (17.37)	742 (17.87)	0.013
Ischemic stroke	1,131 (3.93)	256 (3.25)	875 (4.18)	0.046	272 (3.28)	132 (3.18)	140 (3.37)	0.011
Hypertension	19,003 (65.96)	5,105 (64.83)	13,898 (66.38)	0.033	5,519 (66.46)	2,761 (66.50)	2,758 (66.43)	−0.002
Diabetes	9,449 (32.8)	2,941 (37.35)	6,508 (31.08)	−0.135	2,991 (36.02)	1,502 (36.18)	1,489 (35.86)	−0.007
Congestive heart failure	9,531 (33.08)	3,238 (41.12)	6,293 (30.06)	−0.241	3,202 (38.56)	1,591 (38.32)	1,611 (38.80)	0.01
Chronic pulmonary disease	7,818 (27.13)	2,262 (28.72)	5,556 (26.54)	−0.05	2,440 (29.38)	1,226 (29.53)	1,214 (29.24)	−0.006
Cerebrovascular disease	4,140 (14.37)	910 (11.56)	3,230 (15.43)	0.107	990 (11.92)	499 (12.02)	491 (11.83)	−0.006

### The prognosis of sepsis patients in the low-score group and the high-score group

Table 4 showed the results for the Low-score and High-score groups in both pre- and post-matched cohorts. Before matching, the High-score group had lower 28-day (16.10% vs. 29.54%), 60-day (19.76% vs. 37.07%), 90-day (22.02% vs. 41.26%), and in-hospital (12.77% vs. 22.26%) mortality rates. Post-matching, the High-score group still had lower 28-day (20.23% vs. 25.72%), 60-day (25.31% vs. 32.56%), 90-day (21.76% vs. 40.96%), and in-hospital (16.04% vs. 19.34%) mortality rates compared to the Low-score group.

**Table 4:** Outcomes of the patients with sepsis in the low-score and high-score groups.

Outcomes	Matching	Total	Low-score (< 0.6)	High-score (≥0.6)	*p*
28 day mortality	Before PSM, *n* (%)	5,697 (19.77)	2,326 (29.54)	3,371 (16.10)	< 0.001
	After PSM, *n* (%)	1,908 (22.98)	1,068 (25.72)	840 (20.23)	< 0.001
ICU mortality	Before PSM, *n* (%)	3,270 (11.35)	1,207 (15.33)	2,063 (9.85)	< 0.001
	After PSM, *n* (%)	1,026 (12.36)	542 (13.05)	484 (11.66)	0.053
Hospital mortality	Before PSM, *n* (%)	4,427 (15.37)	1,753 (22.26)	2,674 (12.77)	< 0.001
	After PSM, *n* (%)	1,469 (17.69)	803 (19.34)	666 (16.04)	< 0.001
60 day mortality	Before PSM, *n* (%)	7,056 (24.49)	2,919 (37.07)	4,137 (19.76)	< 0.001
	After PSM, *n* (%)	2,403 (28.94)	1,352 (32.56)	1,051 (25.31)	< 0.001
90 day mortality	Before PSM, *n* (%)	7,759 (26.93)	3,036 (41.26)	4,723 (22.02)	< 0.001
	After PSM, *n* (%)	2,598 (32.69)	1,467 (36.91)	1,131 (28.46)	< 0.001

**Figure 5:**
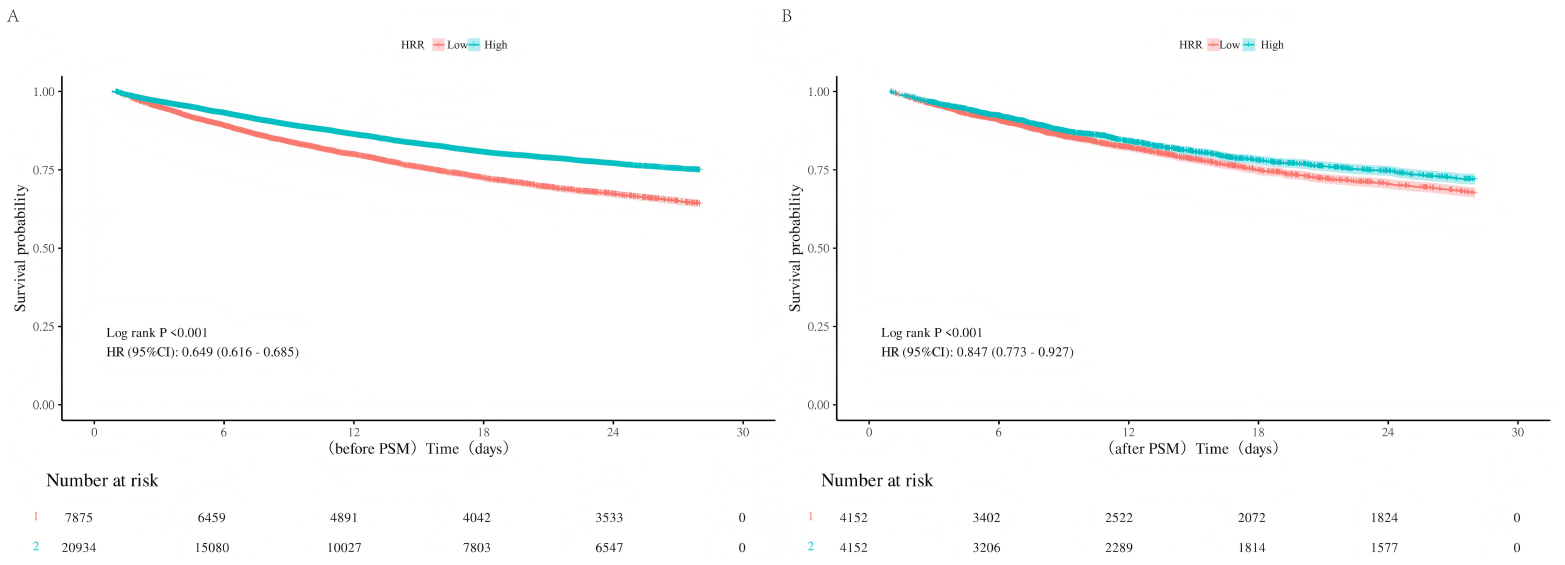
Kaplan–Meier survival curves for 28-day mortality in sepsis patients from the low group and the high group. **(A)** Before PSM. **(B)** After PSM.

The Kaplan–Meier survival curve also indicates that before and after PSM, the 28-day mortality rate in the high-score group was lower (log-rank test: *P* < 0.001; Figure 5). Results from the 60 day and 90 day and in-hospital survival curves were consistent with those from the 28 day results (detailed results are Available in Supplementary Figures 5–7).

### The relationship between HRR and 28-day mortality rate in the pre-matched cohort

Cox proportional hazards regression analysis revealed an independent correlation between HRR and 28-day mortality in sepsis patients (Table 5). Five models were developed for evaluation. When HRR was treated as a continuous variable, it showed an inverse association with 28-day mortality across all five models (*HR*, 0.4; 95% *CI*, 0.32–0.51). The HRR score was also categorized into Low-score (< 0.6) and High-score (≥0.6) groups. Compared to the Low-score group, the High-score group had a 35%, 32%, 36%, 27%, and 27% lower risk of 28-day mortality in models 1–5, respectively. Similar trends were observed when HRR was analyzed as a categorical variable with quintiles, with Q2, Q3, Q4, and Q5 groups showing reduced mortality risks compared to Q1. Additionally, when HRR quintiles were treated as a continuous variable, trend test *P*-values were all below 0.05, reinforcing the correlation's robustness. Notably, the *E*-values were relatively large, further confirming the results' reliability.

**Table 5:** Cox proportional hazard models for 28 day mortality.

Variables	Model 1		Model 2		Model 3		Model 4		Model 5		*E*-value
	*HR* (95% *CI*)	*P*-value	*HR* (95% *CI*)	*P*-value	*HR* (95% *CI*)	*P*-value	*HR* (95% *CI*)	*P*-value	*HR* (95% *CI*)	*P*-value	
HRR as continuous	0.36 (0.32–0.41)	< 0.001	0.43 (0.37–0.49)	< 0.001	0.20 (0.17–0.25)	< 0.001	0.43 (0.34–0.53)	< 0.001	0.40 (0.32–0.51)	< 0.001	4.44
HRR dichotomous											
Low (< 0.6)	Ref	Ref	Ref	Ref	Ref	
High (≥0.6)	0.65 (0.62–0.68)	< 0.001	0.68 (0.64–0.72)	< 0.001	0.64 (0.60–0.69)	< 0.001	0.73 (0.68–0.79)	< 0.001	0.73 (0.68–0.79)	< 0.001	2.08
HRR quartiles											
Q1	Ref	Ref	Ref	Ref	Ref	
Q2	0.73 (0.68–0.79)	< 0.001	0.74 (0.68–0.79)	< 0.001	0.73 (0.67–0.78)	< 0.001	0.79 (0.73–0.85)	< 0.001	0.80 (0.74–0.86)	< 0.001	1.81
Q3	0.64 (0.59–0.69)	< 0.001	0.64 (0.59–0.69)	< 0.001	0.58 (0.53–0.64)	< 0.001	0.69 (0.63–0.75)	< 0.001	0.69 (0.63–0.76)	< 0.001	2.26
Q4	0.58 (0.53–0.63)	< 0.001	0.60 (0.55–0.65)	< 0.001	0.51 (0.46–0.56)	< 0.001	0.66 (0.59–0.73)	< 0.001	0.66 (0.59–0.73)	< 0.001	2.40
Q5	0.59 (0.54–0.64)	< 0.001	0.67 (0.61–0.73)	< 0.001	0.46 (0.40–0.52)	< 0.001	0.66 (0.58–0.75)	< 0.001	0.64 (0.56–0.73)	< 0.001	2.5
P for trend	0.34 (0.30–0.40)	< 0.001	0.41 (0.36–0.48)	< 0.001	0.22 (0.17–0.27)	< 0.001	0.44 (0.35–0.56)	< 0.001	0.42 (0.33–0.53)	< 0.001	4.19

Model 1: crude. Model 2: demographics and vital signs. Model 3: model 2 + laboratory parameters. Model 4: model 2 + treatments and severity scores. Model 5: model 4 + comorbidities.

### The non-linear relationship between HRR and the 28-day mortality risk

**Figure 6:**
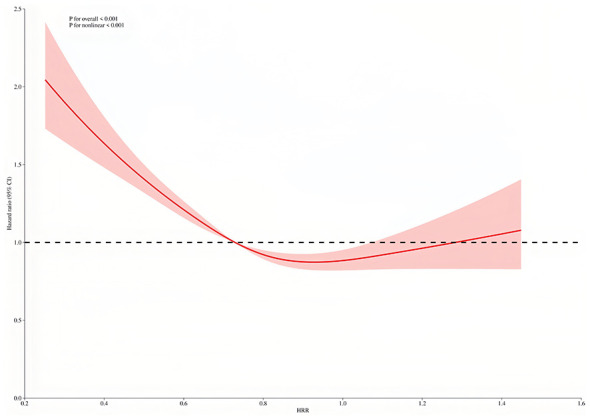
The restricted cubic spline plot of HRR and the 28-day mortality rate of sepsis patients.

Restricted cubic spline analysis revealed a non-linear relationship between HRR and 28-day mortality (*P* for non-linearity < 0.05, Figure 6). The curve showed a steep decline in mortality risk as HRR increased from low values, with the slope flattening at higher HRR levels. A two-segment linear regression model identified an inflection point at *HRR* = 0.78 (Table 6). Below this threshold, each 1-unit increase in HRR was associated with a 74% reduction in 28-day mortality risk (*HR* 0.26, 95% *CI* 0.19–0.38), suggesting that improvements in HRR may be most prognostically meaningful in patients with severe derangements (low hemoglobin and/or high RDW). Above 0.78, the association was attenuated (*HR* 0.64, 95% *CI* 0.40–1.05, *P* = 0.075), indicating a potential ceiling effect where further HRR increases yield diminishing prognostic returns.

**Table 6:** A two-segment linear regression model of HRR and the 28-day mortality rate of sepsis patients.

Outcome	Effect	*P*
Model 1 fitting model by standard linear regression	0.40 (0.32–0.51)	< 0.001
Model 2 fitting model by two-piecewise linear regression		
Inflection point	0.78	
< 0.78	0.26 (0.19–0.38)	< 0.001
≥0.78	0.64 (0.40–1.05)	0.075
*P* for likelihood test		< 0.001

### Prognostic value of HRR and Severity of illness scores

HRR exhibited modest predictive power for 28-day survival, with an AUC of 0.61. Incorporating HRR into the SOFA score enhanced its predictive accuracy for 28-day mortality, boosting the AUC from 0.671 to 0.687 (*P* = 0.005). Similarly, blending HRR with other illness severity metrics also notably improved predictive performance over their standalone use (Table 7).

**Table 7:** The area under the curve (AUC) for predicting 28-day mortality by HRR and HRR combined with other models.

Variables	AUC 95% *CI*	*P*-value
HRR	0.610 (0.602–0.619)	
SOFA	0.671 (0.663–0.679)	
SOFA + HRR	0.687 (0.679–0.695)	< 0.001^a^
SAPSII	0.725 (0.718–0.732)	
SAPSII + HRR	0.733 (0.726–0.741)	< 0.001^b^
OASIS	0.674 (0.666–0.682)	
OASIS + HRR	0.701 (0.693–0.708)	< 0.001^c^
Charlson comorbidity index	0.667 (0.660–0.675)	
Charlson comorbidity index + HRR	0.678 (0.670–0.686)	< 0.001^d^

^a^Represent the statistical significance of the SOFA + HRR in AUC and the SOFA. ^b^Represent the statistical significance of the SAPSII + HRR in AUC and the SAPSII. ^c^Represent the statistical significance of the OASIS + HRR in AUC and the OASIS. ^d^Represent the statistical significance of the Charlson Comorbidity idex + HRR in AUC and the Charlson comorbidity index score.

### Subgroup analysis for in 28-day mortality in patients with sepsis

**Figure 7:**
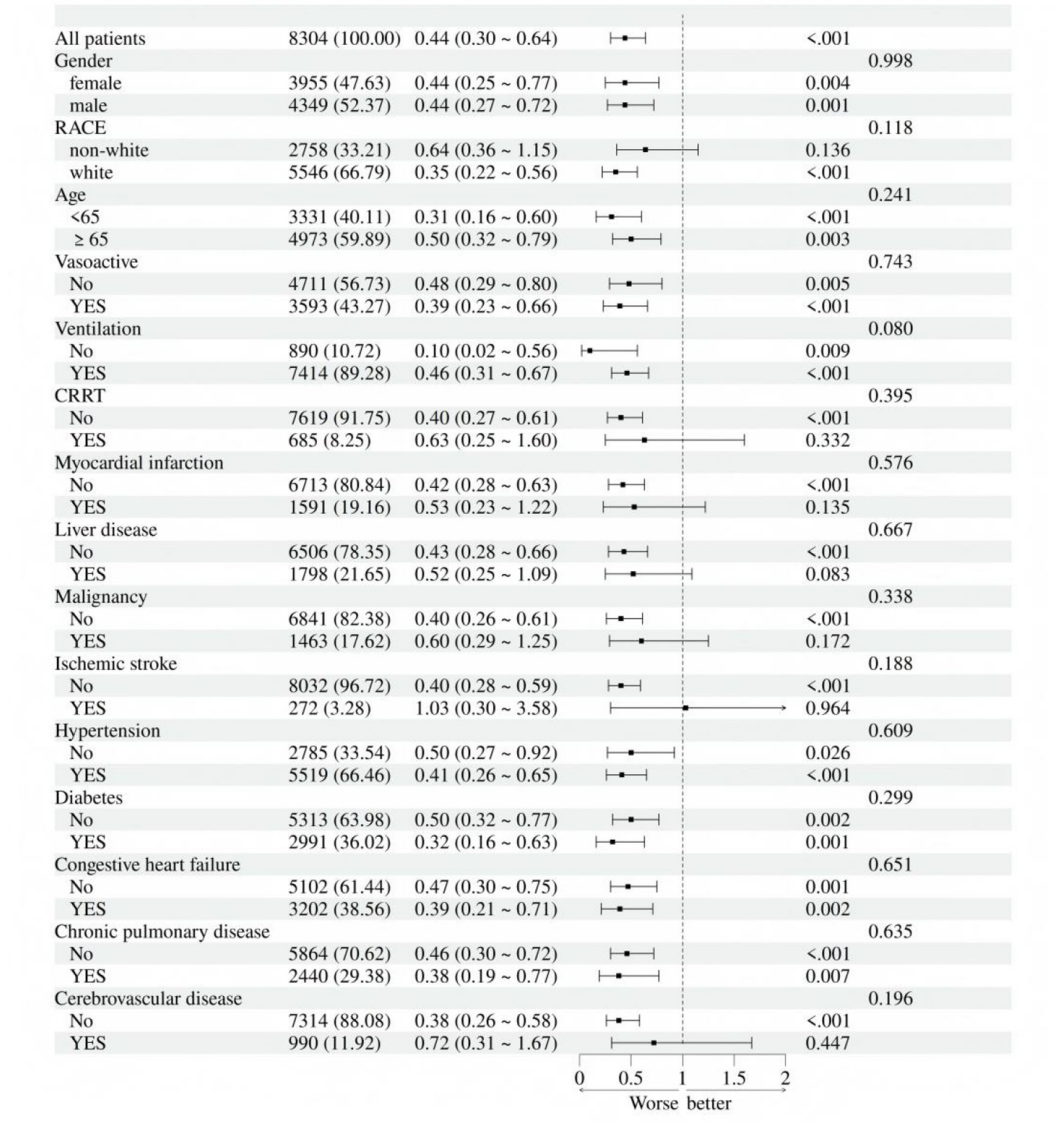
Subgroup analysis of the relationship between HRR and 28-day mortality, as visualized by a forest plot.

Subgroup analyses were carried out to evaluate the role of HRR in predicting 28-day mortality for sepsis patients. The analyses considered various demographic and clinical factors, such as age, gender, ethnicity, comorbidities (such as myocardial infarction, liver disease, malignancy, ischemic stroke, hypertension, diabetes, congestive heart failure, chronic pulmonary disease, and cerebrovascular disease), and treatments (like ventilation, vasopressors, and CRRT). As shown in Figure 7, the results generally linked lower HRR to higher 28-day mortality risks across most subgroups. However, this association wasn't statistically significant in non-white (*P* = 0.136), CRRT (*P* = 0.332), and ischemic stroke (*P* = 0.964) subgroups. Interaction analysis across all subgroups also showed no significant effects.

## Discussion

This study explored the association between HRR and 28-day mortality in sepsis patients using a retrospective cohort from the MIMIC-IV 3.0 database. Results showed that low HRR is associated with higher 28-day mortality. Existing research has shown the connection between inflammatory and nutritional status and adverse outcomes in sepsis patients (17–19). This study emphasizes the independent and combined association of inflammation and nutrition with sepsis prognosis.

Recent meta-analyses highlight the prognostic value of HRR in cancer, where low HRR is associated with poor survival (20). In metastatic renal cancer, low HRR is associated with poor prognosis (21). These findings suggest that low HRR may identify patients at higher risk who warrant closer monitoring. In non-cancer settings, HRR levels are significantly connected to clinical outcomes. In elderly patients with intertrochanteric femur fractures, low HRR is associated with higher AKI risk (22). Liu used the MIMIC database to explore the outcomes of patients with non-traumatic subarachnoid hemorrhage, revealing that a low level of HRR may increase the mortality risk of patients with non-traumatic subarachnoid hemorrhage, emphasizing the need for early intervention in patients with lower HRR, through measures such as improving nutrition and maintaining normal hemoglobin protein levels and controlling inflammation to improve the outcomes of non-traumatic subarachnoid hemorrhage patients (23). Additionally, a decrease in the hemoglobin to red blood cell distribution width ratio in diabetic patients is associated with an increased cardiovascular mortality rate, and a lower HRR is considered an independent prognostic risk factor (24). As a new biomarker, low HRR is associated with mortality in HBV-related decompensated liver cirrhosis patients, showing HRR's potential as a nutritional-inflammation marker for prognosis assessment (25). Our findings reinforce the prognostic relevance of HRR, especially in sepsis patients.

The biological plausibility of HRR as a prognostic marker in sepsis can be understood through several interconnected pathophysiological mechanisms. First, RDW elevation in critical illness reflects erythroid lineage stress driven by systemic inflammation (26). Pro-inflammatory cytokines suppress erythropoietic activity and upregulate hepcidin, leading to functional iron deficiency and impaired erythrocyte maturation (27). Concurrently, oxidative stress damages erythrocyte membranes, further increasing red blood cell size heterogeneity (28). Second, hemoglobin decline in sepsis represents both nutritional depletion and compromised oxygen delivery capacity (29). The HRR integrates these dimensions: a low ratio may indicate concurrent erythropoietic dysfunction and diminished oxygen-carrying reserve, both of which contribute to tissue hypoxia, organ dysfunction, and ultimately mortality.

Sepsis patients are prone to malnutrition, immune-related inflammation, and metabolic disorders (30). Identifying composite biomarkers covering nutrition and inflammation is crucial. Unlike prior inflammatory markers, HRR offers a holistic view of systemic status by combining nutritional and inflammatory indicators, which may make it a useful prognostic marker in this population. The observed association between higher HRR and lower mortality in sepsis patients may reflect multiple underlying pathophysiological processes, as outlined above. HRR, calculated using hemoglobin and RDW, reflects nutritional and inflammatory status. Hemoglobin is a special protein for oxygen transport within red blood cells and its level is closely related to overall nutritional status (31–33). A study evaluating the relationship between nutritional risk indices (such as GNRI) and clinical outcomes found that malnutrition was significantly associated with low hemoglobin levels, and this association was independent of other clinical covariates (34). A 10-year study of Chinese adults found a clear U-shaped relationship between hemoglobin levels and mortality (35). When hemoglobin levels are too low (anemia) or too high, the risk of death significantly increases, suggesting that an optimal but not excessive hemoglobin level may indicate better nutritional status, which could enhance immune function and mitigate the adverse effects of malnutrition. Additionally, RDW reflects red blood cell variability and is elevated in patients with myocardial infarction due to inflammation, oxidative stress, and iron metabolism disorders, leading to impaired erythropoiesis (36). Previous studies have shown that RDW is elevated in sepsis patients and is positively correlated with mortality, indicating that it may indicate the severity of the disease (37, 38). Under such circumstances, the association between higher HRR and improved outcomes may be explained by more favorable underlying pathophysiology, including lower oxidative stress levels, attenuated inflammatory responses, and better nutritional status. Alternatively, HRR may serve as a composite surrogate marker that captures these beneficial characteristics without necessarily being their cause.

While the AUC improvements with HRR addition were statistically significant, their clinical meaningfulness requires careful interpretation. The magnitude of improvement falls below conventional thresholds for clinically important change. However, HRR offers practical advantages: it is derived from routine complete blood count parameters, requires no additional cost, and is immediately available at bedside. In resource-limited settings where lactate or procalcitonin assays are unavailable, HRR may serve as an accessible alternative for early risk stratification. We acknowledge that direct comparison with lactate—the cornerstone of sepsis resuscitation—was precluded by >20% missing data. Future studies should evaluate the clinically practical value beyond statistical significance by combining HRR with comprehensive biomarkers including lactate, NLR and procalcitonin.

Several limitations should be acknowledged. First, this retrospective observational study precludes causal inference; HRR is best interpreted as a surrogate marker reflecting underlying disease severity, inflammatory status, and nutritional state, rather than as an independent causal factor influencing mortality. Second, we excluded variables with >20% missing data, including lactate, CRP, procalcitonin, arterial blood gas parameters, and albumin, which are strongly associated with sepsis severity and mortality. The inability to directly compare HRR with lactate—a cornerstone biomarker in sepsis prognostication—represents limitation in establishing HRR's relative clinical utility. Third, hemoglobin and RDW were excluded from propensity score matching as they directly constitute HRR. While this preserves the exposure-outcome relationship, residual imbalance in underlying hematologic status may persist. Additionally, several variables (RBC count, BUN, liver disease, malignancy, congestive heart failure) showed large standardized mean differences before matching. Although PSM improved balance, residual confounding remains possible given the retrospective observational design. Fourth, while baseline HRR at ICU admission provides robust independent prognostic discrimination, integrating time-varying HRR trajectories may capture evolving pathophysiological states and enhance dynamic risk prediction, warranting prospective validation; similarly, multicenter external validation across diverse populations and healthcare systems remains essential to establish the generalizability of the 0.6 cutoff and facilitate its clinical implementation.

## Conclusion

Our study found that the HRR is associated with short-term and long-term adverse outcomes in sepsis patients. It may serve as a readily available prognostic indicator for risk stratification in sepsis patients.

## Data Availability

The datasets presented in this study can be found in online repositories. The names of the repository/repositories and accession number(s) can be found below: https://mimic.mit.edu/.

## References

[1] Singer M Deutschman CS Seymour CW Shankar-Hari M Annane D Bauer M . The third international consensus definitions for sepsis and septic shock (sepsis-3). JAMA. (2016) 315:801–10. doi: 10.1001/jama.2016.0287 26903338 PMC4968574

[2] Rudd KE Johnson SC Agesa KM Shackelford KA Tsoi D Kievlan DR . Global, regional, and national sepsis incidence and mortality, 1990–2017: analysis for the global burden of disease study. Lancet. (2020) 395:200–11. doi: 10.1016/S0140-6736(19)32989-7 31954465 PMC6970225

[3] Li X Chen Y Yuan Q Zhou H Lu L Guo R. Neutrophil-to-lymphocyte ratio, monocyte-to-lymphocyte ratio, platelet-to-lymphocyte ratio associated with 28-day all-cause mortality in septic patients with coronary artery disease: a retrospective analysis of MIMIC-IV database. BMC Infect Dis. (2024) 24:749. doi: 10.1186/s12879-024-09516-5 39075364 PMC11288105

[4] Zhang Y Peng W Zheng X. The prognostic value of the combined neutrophil-to-lymphocyte ratio (NLR) and neutrophil-to-platelet ratio (NPR) in sepsis. Sci Rep. (2024) 14:15075. doi: 10.1038/s41598-024-64469-8 38956445 PMC11219835

[5] Lai S Li X Cai D Mei C Liang Z. Prognostic value of NPR and CLR-based nomogram modeling in elderly patients with *Acinetobacter baumannii* bloodstream infection. BMC Geriatr. (2025) 25:234. doi: 10.1186/s12877-025-05884-y 40205541 PMC11984014

[6] Sági B Késoi I Vas T Csiky B Nagy J Kovács T. The prognostic role of heart rate recovery after exercise and metabolic syndrome in IGA nephropathy. BMC Nephrol. (2021) 22:390. doi: 10.1186/s12882-021-02596-4 34809611 PMC8609750

[7] Osailan A Metsios GS Rouse PC Ntoumanis N Duda JL Kitas GD . Factors associated with parasympathetic activation following exercise in patients with rheumatoid arthritis: a cross-sectional study. BMC Cardiovasc Disord. (2016) 16:86. doi: 10.1186/s12872-016-0264-9 27165730 PMC4862092

[8] Liu Y Xie Z Wang P Liu F Zhao L Chen C . Relationship between the hemoglobin-to-red cell distribution width ratio and post-stroke cognitive impairment: a prospective study. Front Aging Neurosci. (2025) 17:1552956. doi: 10.3389/fnagi.2025.1552956 40370755 PMC12075231

[9] Johnson AEW Bulgarelli L Shen L Gayles A Shammout A Horng S . MIMIC-IV, a freely accessible electronic health record dataset. Sci Data. (2023) 10:1. doi: 10.1038/s41597-023-01945-2 36596836 PMC9810617

[10] Chen J Wu Y Zhao H Ruan G Qin S. Ratio of hemoglobin to red cell distribution width: an inflammatory predictor of survival in aids-related DLBCL. Front Immunol. (2024) 15:1354325. doi: 10.3389/fimmu.2024.1354325 38426083 PMC10901994

[11] Kontopantelis E White IR Sperrin M Buchan I. Outcome-sensitive multiple imputation: a simulation study. BMC Med Res Methodol. (2017) 17:2. doi: 10.1186/s12874-016-0281-5 28068910 PMC5220613

[12] Kim JH. Multicollinearity and misleading statistical results. Korean J Anesthesiol. (2019) 72:558–69. doi: 10.4097/kja.19087 31304696 PMC6900425

[13] Camp RL Dolled-Filhart M Rimm DL. X-Tile: a new bio-informatics tool for biomarker assessment and outcome-based cut-point optimization. Clin Cancer Res. (2004) 10:7252–9. doi: 10.1158/1078-0432.CCR-04-0713 15534099

[14] Austin PC. An introduction to propensity score methods for reducing the effects of confounding in observational studies. Multivariate Behav Res. (2011) 46:399–424. doi: 10.1080/00273171.2011.568786 21818162 PMC3144483

[15] Yang JY Parkins MD Canakis A Aroniadis OC Yadav D Dixon RE . Outcomes of Covid-19 among hospitalized health care workers in North America. JAMA Netw Open. (2021) 4:e2035699. doi: 10.1001/jamanetworkopen.2020.35699 33507259 PMC7844592

[16] Haneuse S VanderWeele TJ Arterburn D. Using the E-value to assess the potential effect of unmeasured confounding in observational studies. JAMA. (2019) 321:602–3. doi: 10.1001/jama.2018.21554 30676631

[17] Toscano A Bellone F Maggio N Cinquegrani M Spadaro F Bueti FM . Unlocking the predictive power of nutritional scores in septic patients. Nutrients (2025) 17:545. doi: 10.3390/nu17030545 39940402 PMC11820051

[18] Pei C Dong Y Song N. Association between advanced lung cancer inflammation index and mortality in critically ill septic patients: analysis of the MIMIC-IV database. BMC Infect Dis. (2025) 25:747. doi: 10.1186/s12879-025-11116-w 40413403 PMC12103769

[19] Saridaş A Çetinkaya R. The prognostic value of the Cally index in sepsis: a composite biomarker reflecting inflammation, nutrition, and immunity. Diagnostics. (2025) 15:1026. doi: 10.3390/diagnostics15081026 40310418 PMC12025508

[20] Coradduzza D Medici S Chessa C Zinellu A Madonia M Angius A . Assessing the predictive power of the hemoglobin/red cell distribution width ratio in cancer: a systematic review and future directions. Medicina. (2023) 59:2124. doi: 10.3390/medicina59122124 38138227 PMC10744746

[21] Yilmaz H Yilmaz A Demirag G. Prognostic significance of hemoglobin-to-red cell distribution width ratio in patients with metastatic renal cancer. Future Oncol. (2021) 17:3853–64. doi: 10.2217/fon-2021-0040 34382414

[22] Yuan X Zeng W Wang H Shu G Wu C Nie M . Predictive value of the early postoperative hemoglobin-to-red blood cell distribution width ratio for acute kidney injury in elderly intertrochanteric fracture patients. BMC Musculoskelet Disord. (2024) 25:630. doi: 10.1186/s12891-024-07745-y 39113005 PMC11308471

[23] Liu J Wang J. Association between hemoglobin-to-red blood cell distribution width ratio and hospital mortality in patients with non-traumatic subarachnoid hemorrhage. Front Neurol. (2023) 14:1180912. doi: 10.3389/fneur.2023.1180912 37388548 PMC10303799

[24] Deng J Wu W Zhang Z Ma X Chen C Huang Y . Association between reduced hemoglobin-to-red cell distribution width ratio and elevated cardiovascular mortality in patients with diabetes: insights from the national health and nutrition examination study, 1999–2018. Clin Hemorheol Microcirc. (2025) 89:69–81. doi: 10.3233/CH-242209 39439352

[25] Yu Z Zhang T Shen J. Low hemoglobin-to-red cell distribution width ratio is associated with mortality in patients with HBV-related decompensated cirrhosis. Biomed Res Int. (2022) 2022:5754790. doi: 10.1155/2022/5754790 35198637 PMC8860564

[26] Crook JM Horgas AL Yoon SL Grundmann O Johnson-Mallard V. Vitamin C plasma levels associated with inflammatory biomarkers, CRP and RDW: results from the NHANES 2003–2006 surveys. Nutrients (2022) 14:1254. doi: 10.3390/nu14061254 35334908 PMC8950002

[27] Quintana-Castanedo L Maseda R Pérez-Conde I Butta N Monzón-Manzano E Acuña-Butta P . Interplay between iron metabolism, inflammation, and EPO-ERFE-hepcidin axis in RDEB-associated chronic anemia. Blood Adv. (2025) 9:2321–35. doi: 10.1182/bloodadvances.2024015271 40036737 PMC12127647

[28] Bai Y Chen J Zhang S Xu G Mao Z Ding Y . Inflammation-responsive cell membrane-camouflaged nanoparticles against liver fibrosis via regulating endoplasmic reticulum stress and oxidative stress. Adv Mater. (2024) 36:e2310443. doi: 10.1002/adma.202310443 38372054

[29] Kuang L Zhu Y Zhang J Wu Y Tian K Chen X . A novel cross-linked haemoglobin-based oxygen carrier is beneficial to sepsis in rats. Artif Cells Nanomed Biotechnol. (2019) 47:1496–504. doi: 10.1080/21691401.2019.1602049 30983419

[30] Greenberg JA Hohmann SF James BD Shah RC Hall JB Kress JP . Hospital volume of immunosuppressed patients with sepsis and sepsis mortality. Ann Am Thorac Soc. (2018) 15:962–9. doi: 10.1513/AnnalsATS.201710-819OC 29856657 PMC6322036

[31] Mutumba R Mbabazi J Pesu H Greibe E Olsen MF Briend A . Micronutrient status and other correlates of hemoglobin among children with stunting: a cross-sectional study in Uganda. Nutrients (2023) 15:3785. doi: 10.3390/nu15173785 37686816 PMC10489905

[32] Steinmeyer Z Delpierre C Soriano G Steinmeyer A Ysebaert L Balardy L . Hemoglobin concentration; a pathway to frailty. BMC Geriatr. (2020) 20:202. doi: 10.1186/s12877-020-01597-6 32527230 PMC7291509

[33] Makurat J Friedrich H Kuong K Wieringa FT Chamnan C Krawinkel MB. Nutritional and micronutrient status of female workers in a garment factory in Cambodia. Nutrients (2016) 8:694. doi: 10.3390/nu8110694 27827854 PMC5133081

[34] Huang Y Wang X Li Z Yin X. A novel nutritional inflammation index for predicting mortality in acute ischemic stroke patients: insights into advanced lung cancer inflammation index from the medical information mart for intensive care-IV database. Front Nutr. (2024) 11:1408372. doi: 10.3389/fnut.2024.1408372 39036488 PMC11257925

[35] Shi Z Zhen S Zhou Y Taylor AW. Hb level, iron intake and mortality in Chinese adults: a 10-year follow-up study. Br J Nutr. (2017) 117:572–81. doi: 10.1017/S000711451700040X 28382896

[36] Guo T Qin Z He D. Acute myocardial infarction (AMI) as the effect modifiers to modify the association between red blood cell distribution width (RDW) and mortality in critically ill patients with stroke. Front Med. (2022) 9:754979. doi: 10.3389/fmed.2022.754979 35559346 PMC9086673

[37] Wang TH Hsu YC. Red cell distribution width as a prognostic factor and its comparison with lactate in patients with sepsis. Diagnostics. (2021) 11:1474. doi: 10.3390/diagnostics11081474 34441408 PMC8394551

[38] Wu YC Chen HH Chao WC. Association between red blood cell distribution width and 30-day mortality in critically ill septic patients: a propensity score-matched study. J Intensive Care. (2024) 12:34. doi: 10.1186/s40560-024-00747-x 39294760 PMC11409593

