# Supplementary figures and images for: Assessment of hemoglobin-to-red cell distribution width ratio to predict all-cause mortality in patients with sepsis: a retrospective cohort study from the MIMIC-IV database
Source: Front Med (Lausanne). 2026 Jul 13;13:1836373. doi: 10.3389/fmed.2026.1836373 (PMC13402431; doi:10.3389/fmed.2026.1836373)

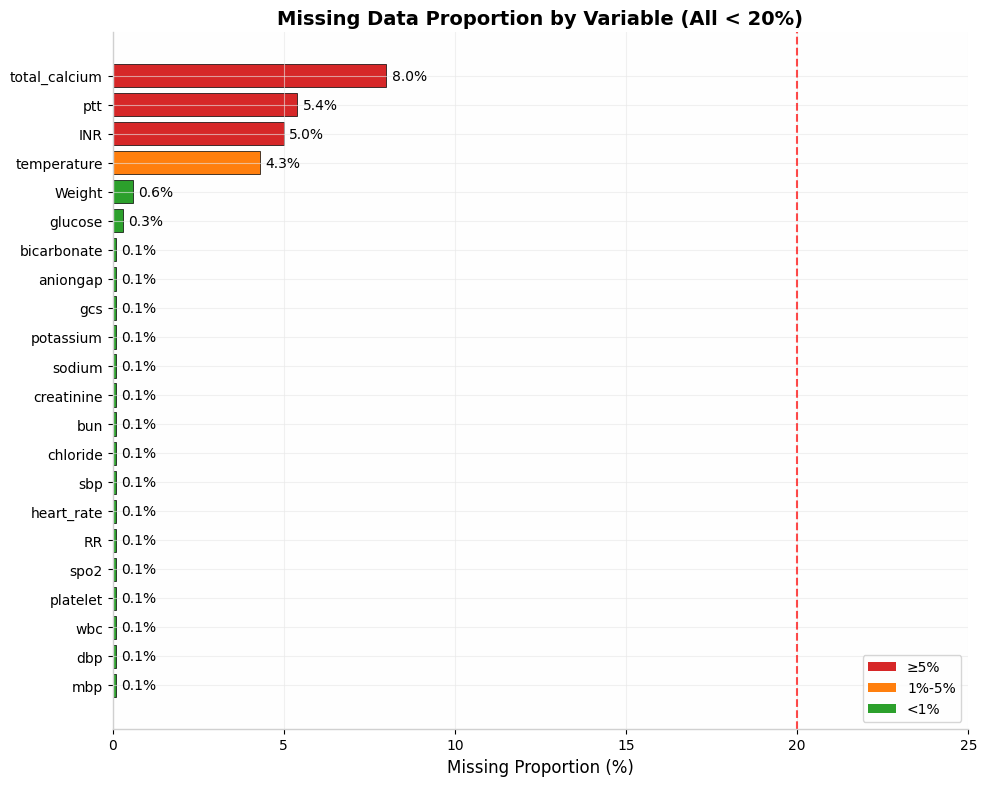

Supplement: Supplementary file 1 [file Image_1.PNG]

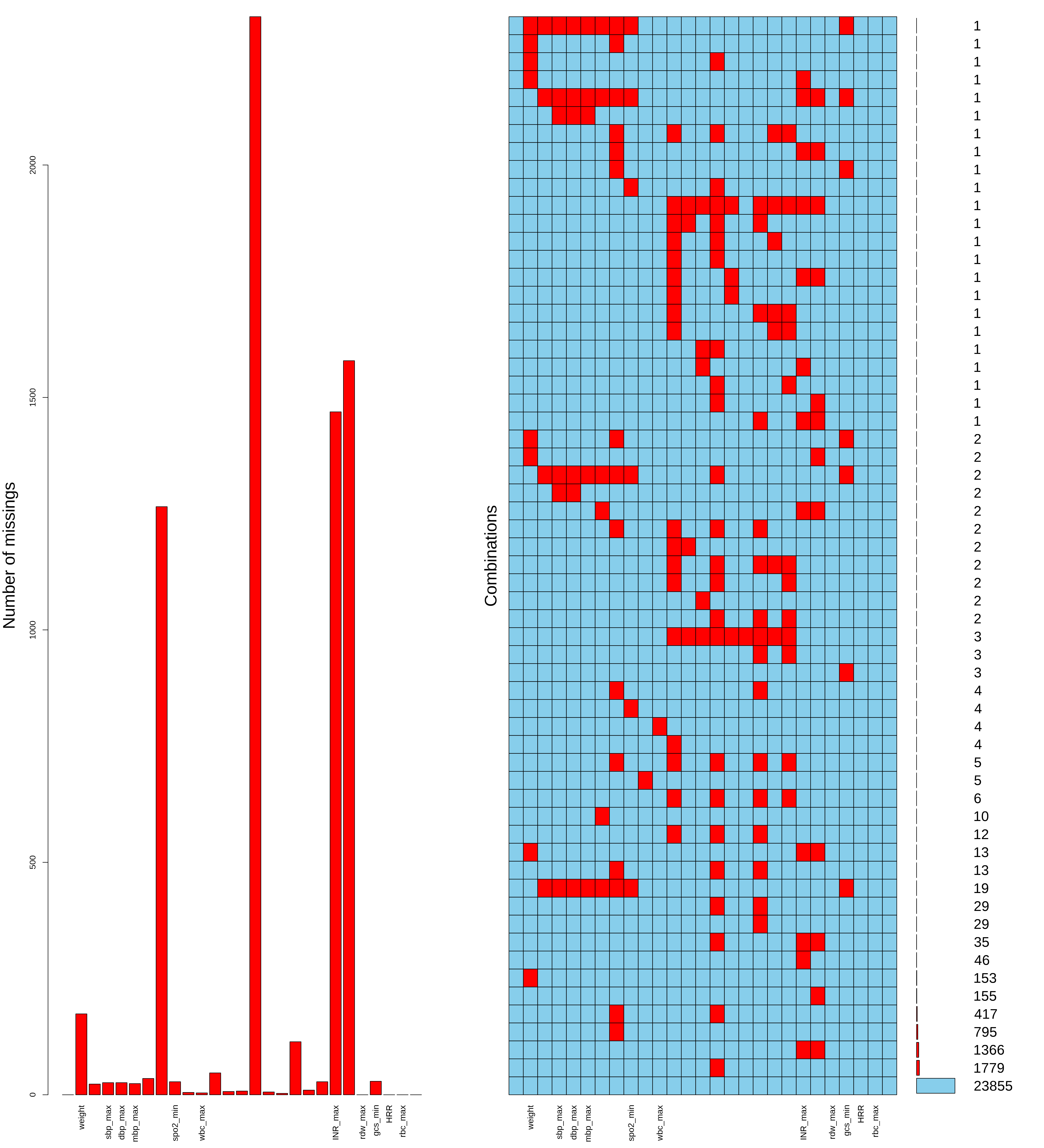

Supplement: Supplementary file 2 [file Image_2.PNG]

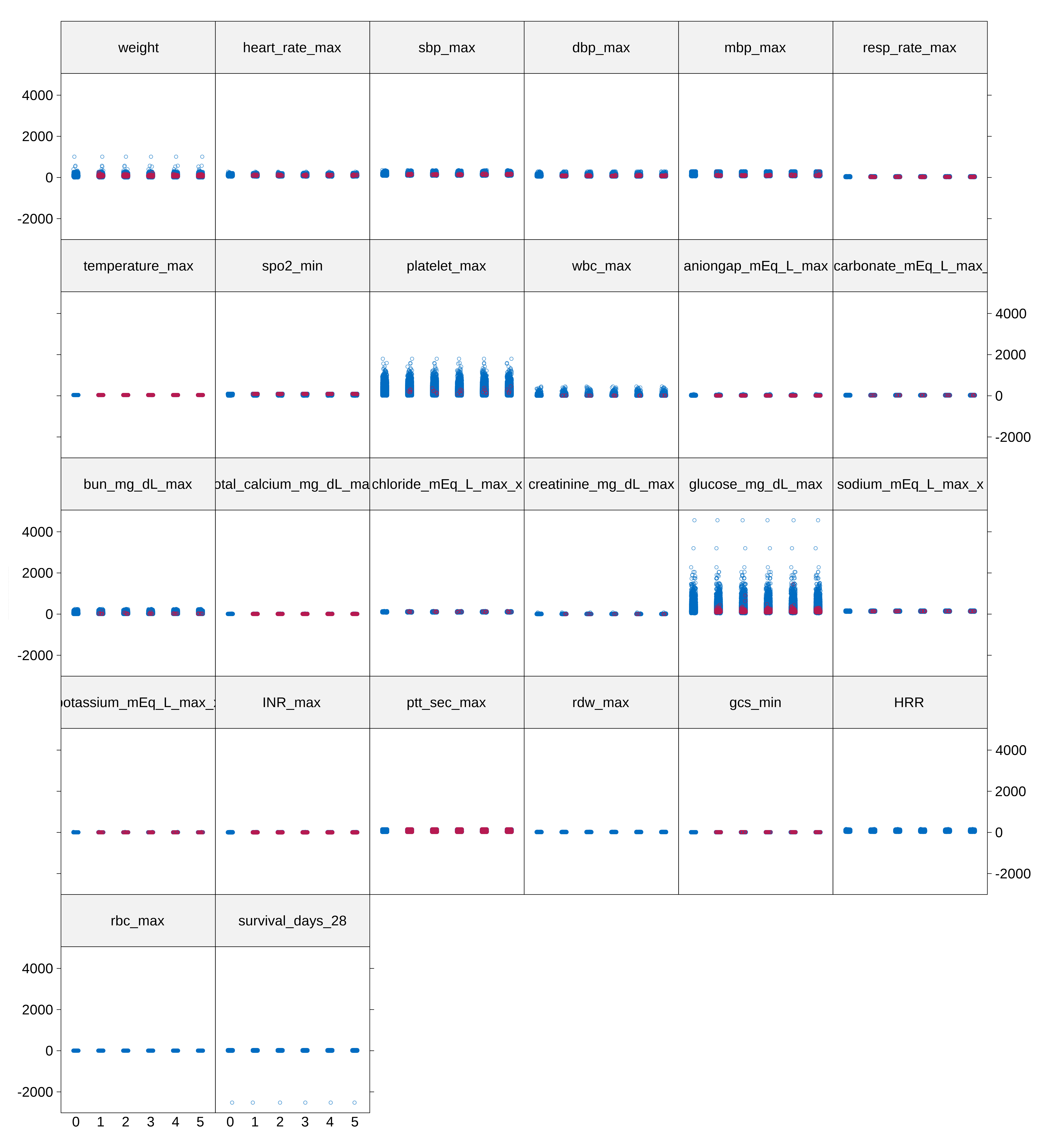

Supplement: Supplementary file 3 [file Image_3.PNG]

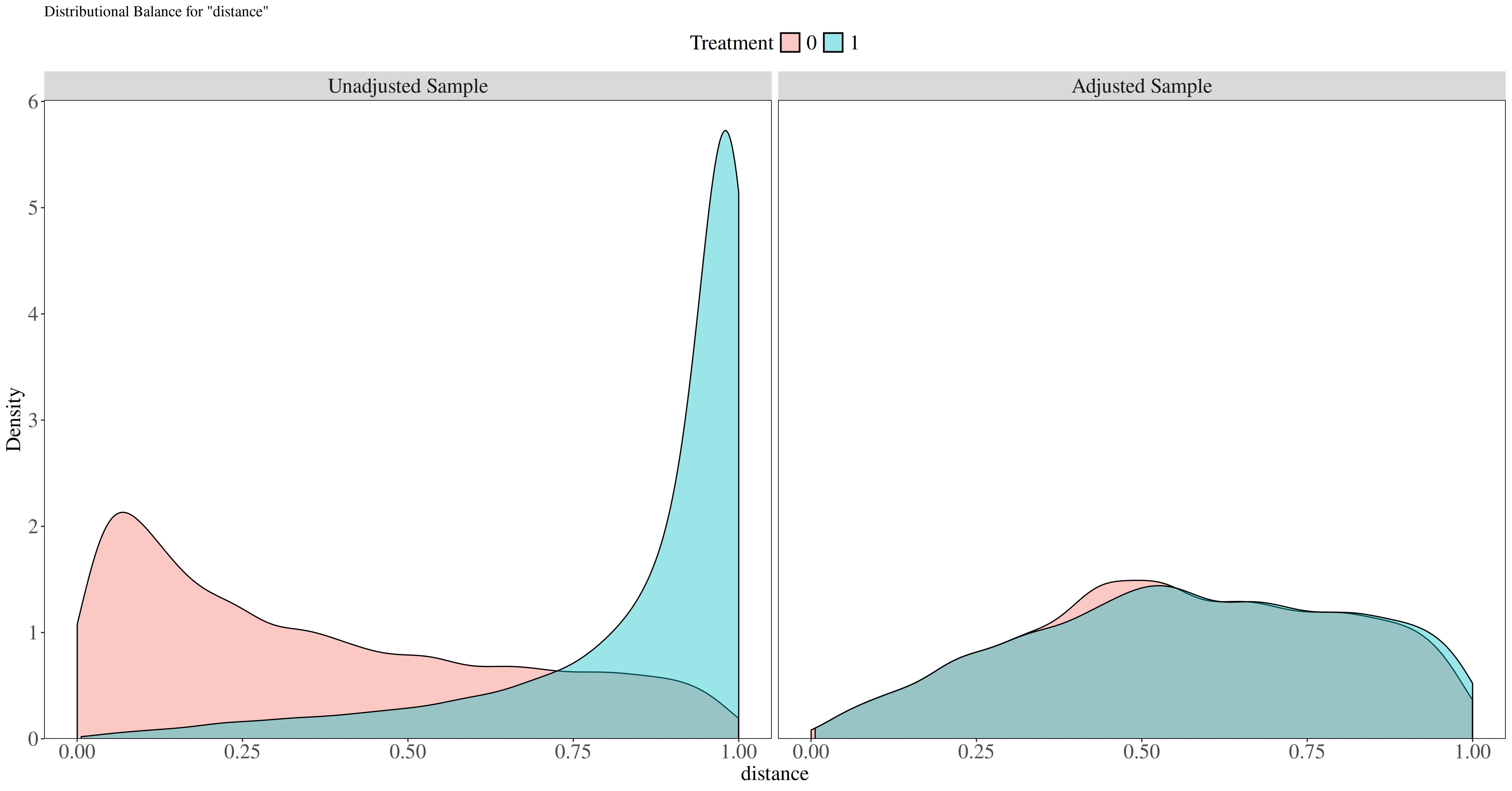

Supplement: Supplementary file 4 [file Image_4.JPEG]

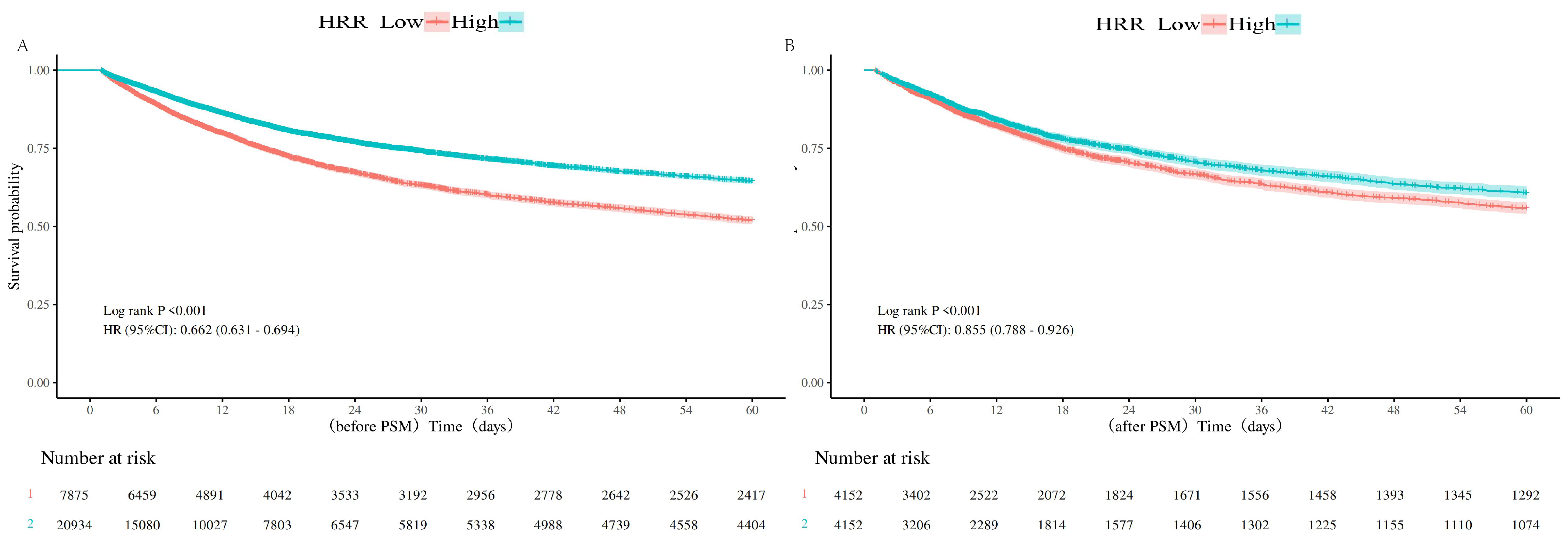

Supplement: Supplementary file 5 [file Image_5.JPEG]

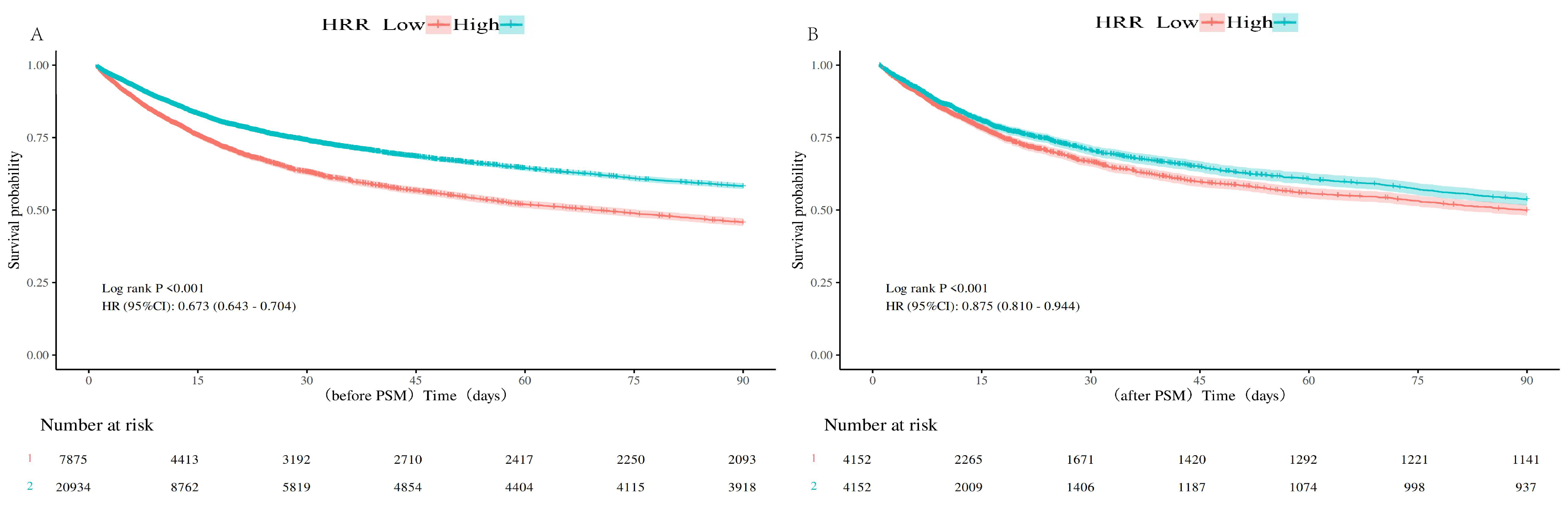

Supplement: Supplementary file 6 [file Image_6.JPEG]

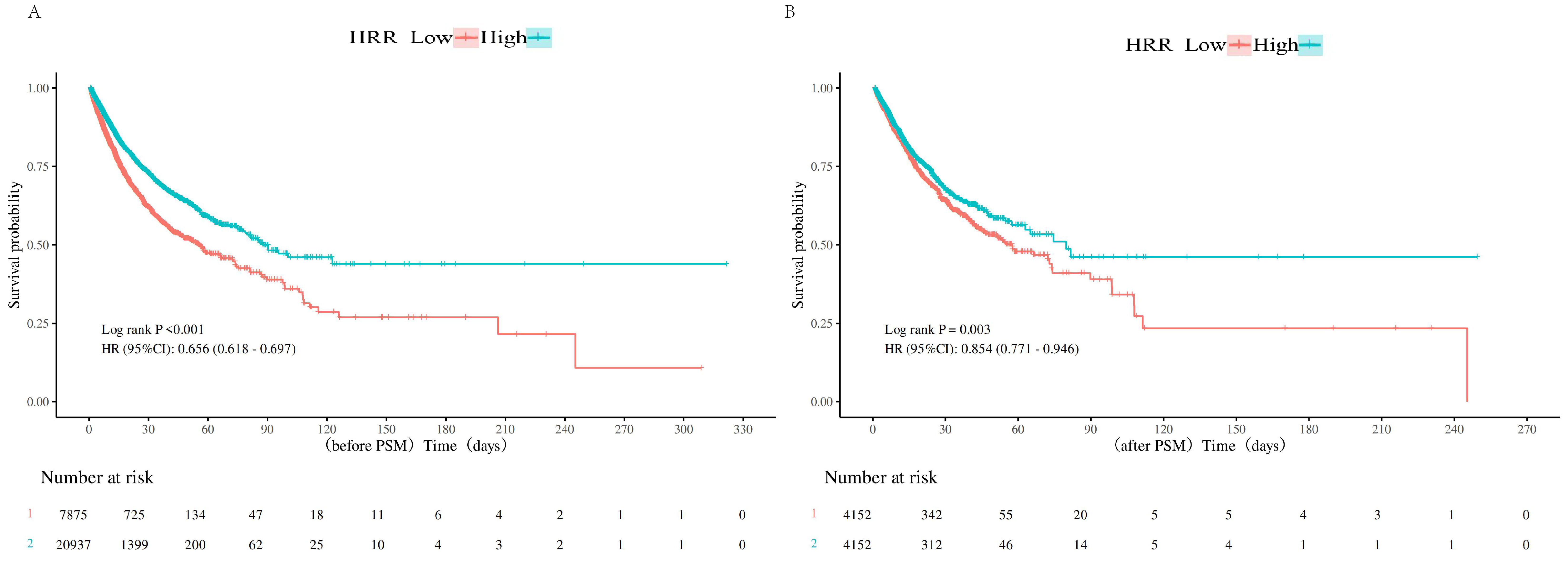

Supplement: Supplementary file 7 [file Image_7.JPEG]
